# Do vitamin A deficiency and undernutrition still matter?

**Published:** 2013

**Authors:** Klaus Kraemer, Clare Gilbert

**Affiliations:** Director: Sight and Life, Basel, Switzerland, Adjunct Associate Professor: Johns Hopkins Bloomberg School of Public Health, Baltimore, USA.; Co-director: International Centre for Eye Health, Disability Group, London School of Hygiene and Tropical Medicine, London, UK.

**Figure:**
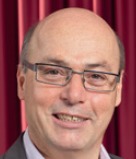
Klaus Kraemer

**Figure:**
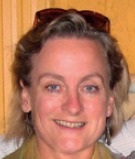
Clare Gilbert

Children grow and develop well when they have access to affordable, diverse, nutrient-rich food, appropriate maternal and child care, adequate health services and a healthy environment including safe water, sanitation and good hygiene.

Children can become undernourished – therefore failing to grow and thrive – for a variety of reasons. The immediate causes of undernutrition are:

- **inadequate dietary intake**, whether as a result of poor maternal diet before, during and after pregnancy, sub-optimal breastfeeding, inadequate complementary foods during weaning or insufficient nutrient-rich foods during early childhood
- **disease**, including parasitic infections, diarrhoeal disease, or other infections such as measles.

Underlying these causes are factors such as household food insecurity (due to poverty or other reasons), inadequate care and feeding practices, unhealthy household environments and inadequate health services.^1^ How these factors inter-relate with each other is shown on page 64.

Being undernourished for a long time can lead to stunting. In addition to its most obvious effects on stature (height), stunting has implications for the health and development of children, including their ability to learn. It can also lead to an increased risk of chronic diseases, such as heart disease or diabetes, in adulthood.

Although stunting is declining, the rate of decline is too slow. There are now major global initiatives in place to improve the nutritional status of young children – the group most vulnerable to undernutrition and in whom the effects of undernutrition are greatest.

**Figure:**
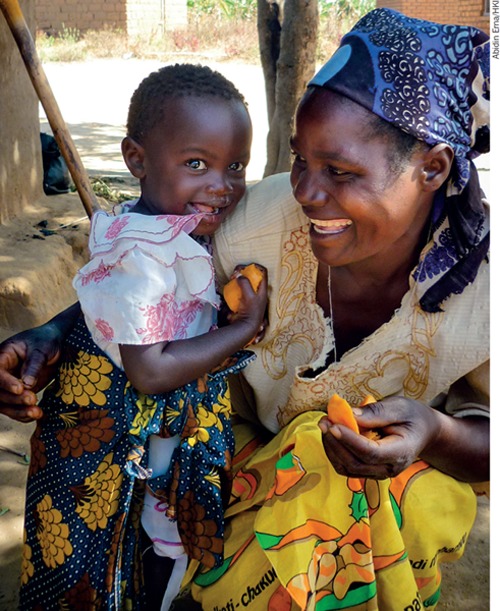
Having enough nutrient-rich food to eat prevents stunting, blindness, and death in young children

Two of the main initiatives are the Scaling Up Nutrition (SUN) movement and the 1,000 Days Partnership, which focuses on the period of that encompasses pregnancy and the first two years of the child's life. These initiatives have similar aims. Broadly, these are:

- to improve women's nutrition before, during and after pregnancy to promote intrauterine growth and improve the quality of breast milk
- to promote and support exclusive breastfeeding for the first 6 months of a child's life followed by continued breastfeeding together with the introduction of safe and appropriate complementary feeding for the next 18 months and beyond
- to ensure children get the vitamins and minerals they need, whether through better dietary choices, food fortification or micronutrient supplementation
- to treat malnutrition with appropriate nutritional interventions.

Improving the availability of affordable, nutritious foods requires a broad approach, encompassing all the farmers, businesses, institutions and processes (such as supply chains) which produce, process and make foods available to communities.

**Figure:**
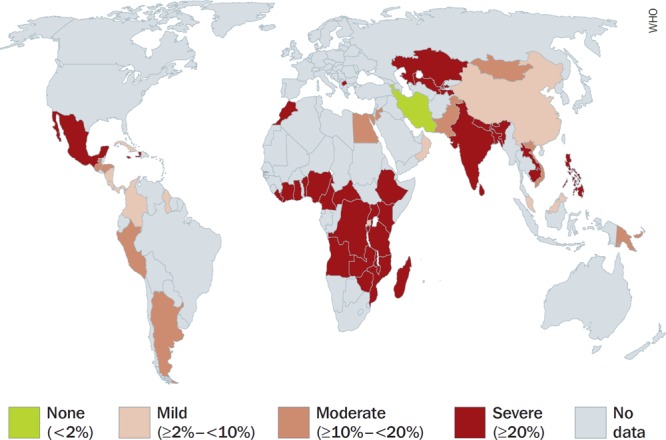
Figure 1. Global extent of vitamin A deficiency as defined by prevalence of serum reinol l <0.70 µmol/l in preschool children^3^

## Vitamin A deficiency remains a problem

Most children who suffer from malnutrition and stunting are deficient in many micronutrients, such as the B vitamins, vitamin D, iron, iodine and zinc. Stunted children are also usually deficient in vitamin A, which places them at increased risk of blindness and death.

- **Blindness**. Vitamin A deficiency can result in xerophthalmia (‘dry eyes’), which in its most severe forms results in irreversible blindness (pages 66–67). Xerophthalmia, in the form of night blindness, and low levels of retinol (the form of vitamin A found in the blood), are both indicators of vitamin A deficiency.
- **Death**. In children, the link between vitamin A deficiency and death is so strong that mortality rates in children under 5 years are now taken to be a ‘surrogate’ indicator of vitamin A deficiency. Vitamin A deficiency is considered to be a public health issue in countries with mortality rates in children under 5 years of ≥50 deaths per 1,000 live births. In sub-Saharan Africa, 40 countries have mortality rates in under-5s above this level; of these, 37 have mortality rates that are twice as high (over 100 deaths per 1,000).^2^

Although great strides have been made to address vitamin A deficiency in children, it is clear from these data that there are still many countries where vitamin A deficiency remains a problem. Worldwide, in populations at risk of vitamin A deficiency, one in three preschool-aged children is thought to be deficient in vitamin A with the greatest burden in Africa and Southeast Asia (Figure 1).^3^

In the short term, vitamin A supplementation is the most effective way to reduce vitamin A deficiency and child mortality (see page 70). Doing something about vitamin A deficiency on its own, however, will not deal with the larger problem of undernutrition and deficiency of other micronutrients essential for growth, health and educational development.

This is why, in this issue of the *Community Eye Health Journal*, we suggest that vitamin A deficiency must be addressed – not just with supplementation – but also by working with mothers to address the immediate and underlying causes of chronic undernutrition. This will improve their children's health and diet and therefore also their general nutrition.

In particular, we should encourage improved hand washing practices and work with families to overcome customs associated with inadequate complementary or weaning foods.

‘There are still many countries in which vitamin A deficiency remains a problem’

Vitamin A supplementation – a specific, targeted intervention delivered by health workers – remains an important and effective strategy for reducing vitamin A deficiency. Many countries are achieving high coverage, but even in these countries, many infants and children living in poor, rural communities are not being reached. This issue discusses some of the ways in which coverage can be improved and highlights the successes achieved in Burkina Faso.

**Figure:**
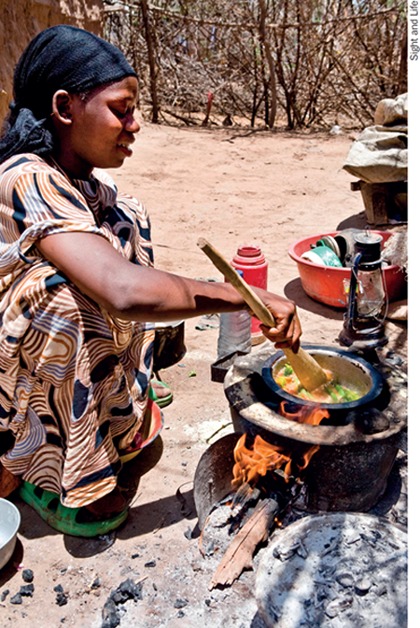
Providing mothers and carers with the knowledge and skills to prepare nutrient-rich foods can reduce undernutrition and vitamin A deficiency, but sometimes more is needed

As eye care professionals, we can do a lot to inform and educate communities about nutrition in general and how families can improve the diet of young children, thereby also preventing them from becoming vitamin A deficient. This issue gives some practical examples of what you can do either in the clinic or during outreach and includes advice on how to manage a child with xerophthalmia and what urgent action is needed to reduce the risk of blindness and death.

Chronic undernutrition affects communities, not just individuals. It is therefore important to remember that when we see a child with xerophthalmia, there are likely to be many more children affected by the condition in his or her community. Many of these children, although vitamin A deficient, will not show the eye signs.

Vitamin A deficiency and undernutrition still matter. As eye health workers, we have a responsibility to do what we can, and also to alert those responsible for child health if we suspect that a particular community is suffering from chronic undernutrition – as they are likely to be vitamin A deficient too.
